# Relationship between Demographic Factors and Violence during Pregnancy in Iran: A Meta-Analysis Study

**Published:** 2018-10

**Authors:** Tahereh Bahmani, Koroush Sayehmiri, Salman Daliri, Arezoo Karimi

**Affiliations:** 1 Student Research Committee, School of Health, Ilam University of Medical Sciences, Ilam, Iran.; 2 Prevention Center of Social Mental Injuries, School of Medicine, Ilam University of Medical Sciences, Ilam, Iran.

**Keywords:** *Demographic Factors*, *Domestic Violence*, *Iran*, *Meta-Analysis*, *Pregnancy*

## Abstract

**Objective:** Domestic violence is the most common form of violence against women and a major health problem worldwide. The aim of this study was to examine the relationship between demographic factors and domestic violence during pregnancy through meta-analysis.

**Method** **:** This meta-analysis study was conducted in Iran. All the articles published during 2001 up to Jun 2018 were extracted independently by 2 trained investigators from domestic and foreign databases including, Science Medlib, SID, Web of Science, PubMed, Science Direct, Irandoc, Medline, Scopus, Magiran, and Google Scholar with keywords and their compounds. The results of studies pooled using the random effects model Cochran and I2 tests were used to check heterogeneity. Data were analyzed using Stata Ver. 11.2.

**Results: **A total of 28 articles with the sample size of 15 020 people were included in the study. The findings of the meta-analysis showed that low level of maternal education (OR:1.68) (CI 95%:1.15, 2.46), low education level of the spouse (OR:1.73) (CI 95%:1.31, 2.29), unemployment of the husband (OR:1.61) (CI 95%: 1.05, 2.48), and smoking of the husband (OR:2.51) (CI 95%: 1.64, 3.84) were important factors in the increase in domestic violence during pregnancy. Having 3 children or fewer (OR: 0.30) (CI 95%: 0.16, 0.56) and enough and regular visits to physicians to receive adequate prenatal care (OR: 0.31) (CI 95%: 0.16, 0.57) were deterrent for violence during pregnancy.

**Conclusion: **Based on our findings, level of education, unemployment, prenatal care, smoking, and number of children are associated with violence during pregnancy. Thus, paying attention to these factors and controlling them can reduce violence during pregnancy and its adverse consequences.

Domestic violence is a pattern of behaviors which involves harassment, intimidation, and threatening behavior, violence, or other abuse by one person against another in a domestic setting, such as in marriage or cohabitation that includes physical, sexual, psychological, and economic abuse and verbal threats (1). Domestic violence is the most common form of violence experienced by women. This phenomenon is one of the world`s most pressing health problems (2). Violence against women occurs in all countries of the world. It is a social, legal, and health problem that occurs even during pregnancy and may potentially threat the lives of both the mother and the fetus (3, 4). 

Domestic violence may start or get worse during pregnancy (5). 

According to the World Health Organization report, 45% of women suffer from domestic violence. Based on this report, prevalence of domestic violence during pregnancy in various communities has been reported to be 9%-21% (6). Nasir et al. have measured violence during pregnancy in developing countries and found it to be 4%-28% (7). Jahanfar et al. have estimated the prevalence of domestic violence in pregnant women referring to hospitals affiliated to Tehran University of Medical Sciences to be 6.60% (8). 

In the study of Karimi et al., the prevalence of physical violence during pregnancy was estimated to be 18% in the world and 23% in Iran and the prevalence of psychological violence 38% and 44% in the world and Iran, respectively (9). 

In the study of Bazyar et al., the prevalence of sexual violence during pregnancy was 17% in the world and 28% in Iran (10).

Many reasons are associated with the occurrence of domestic violence against women, especially during pregnancy, which can be affected by social, economic, and cultural factors. The main causes of domestic violence are maternal age, maternal education, husband's education, having more children, age of marriage younger than 20 years (11, 12), unintended pregnancies, women's' jobs, unemployment of the husband, smoking of the husband (11, 13), low family income (12), and personality traits of the husband. Thus, many risks, including physical injury, abdominal and uterus trauma, and mental health problems threaten pregnant women under domestic violence. In cases where violence occurs frequently, the number of women who visit health centers to receive prenatal care decreases or they would be prevented from referring to these centers (14). As a result, these women attend to health centers with delay to receive prenatal care which results in incidence of severe adverse neonatal outcomes, including preterm birth and low birth weight (15, 16). 

Based on the results of these studies, the prevalence of domestic violence during pregnancy in Iran, in comparison to the global average, is higher and its prevalence rate is far above which causes adverse effects on individuals, families, and societies. Domestic violence is associated with many demographic characteristics, including age, education, and socioeconomic status. In this regard, various studies in different regions of the country have investigated this field and each of them has reported different rates of violence during pregnancy. Therefore, this study aimed to investigate the relationship between demographic factors and incidences of domestic violence during pregnancy in Iran by a meta-analysis to identify factors associated with this phenomenon and control them to reduce the incidence of this social and health problem.

## Materials and Methods

***Search Strategy***

This was a systematic review and meta-analysis study in the context of demographic factors, with special attention to the incidence of domestic violence during pregnancy in Iran. In this study, all published articles since the beginning of 2001 to the end of Jun 2018 in domestic and foreign databases, such as Global Medical Article Limberly (Medlib), Scopus, Web of Science, PubMed, Cochrane Library, Science Direct, Google Scholar, Irandoc, Iranian Journal Database (Magiran), Iranian Biomedical Journal (IranMedex), and Scientific Information Databases (SID), were extracted. Searching for articles was done using the following keywords, independently or in combined forms: Domestic Violence, Violence during pregnancy, Demographic factor, Iran MeSh combined with the operators "OR" vs. "AND". 

***Study Selection and Data Extraction***

All articles that have examined domestic violence during pregnancy and its influencing factors were included without restrictions. To reduce errors in data collection, data extraction was performed independently by 2 investigators and the results of repetitive searches were omitted. In case of rejection of a paper by each investigator, the reason of rejection was required, and in case of any discrepancies between the 2 investigators, a third investigator was asked to evaluate the paper. To check the quality of the articles, STROBE checklist was used (Strengthening the reporting of observational studies in epidemiology) (17). This checklist has 22 sections and is rated based on the importance of each part. The total score of the checklist is 30, and the minimum acceptable score is 15.

Inclusion criteria were as follow: (1) studies in English and Persian languages in Iran which are about domestic violence during pregnancy and factors affecting it; (2) studies that have examined and involved domestic violence during pregnancy in the form of total violence or domestic violence and its dimensions, including physical, emotional, psychological, sexual, economic, social and verbal, or speech violence; (3) being a quantitative research; (4) the study population had to consist of pregnant and postpartum women; (5) The studies had to deal with demographic risk factors affecting the incidence of domestic violence against women in one of the 2 levels of victims of domestic violence (women) or violators (men); (6) those observational and descriptive studies (cross sectional, cohort, case-control) that examined domestic violence against women during pregnancy and postprocess quantification and had received points higher than 20 were included in the research. Exclusion criteria were as follow: studies which discussed violence during pregnancy and its influencing factors but their data were reported as mean and standard deviation and those studies with insufficient data or without the required information and lack of the inclusion criteria for the study were excluded. 

Required information of the used articles in the meta-analysis process was obtained by a preset checklist that consisted of the researcher’s name, year of study, place of study, sample size, type of study, and the required data in the agreement table to calculate odds ratios and (ORs) and 95% confidence intervals (CIs) of influencing demographic factors on violence against women during pregnancy in 2 levels of victims of domestic violence (women) and violators (men). The studied variables were as follow: maternal age equal to or less than 25 years compared to older than 25 years; marriageable age equal and less than 18 years compared to older than 18 years; 3 children or less compared to more than 3 children; housewife mothers compared to employed ones; unintended pregnancy versus wanted pregnancy; pregnancy care more than 6 months compared to equal or less than 6 months; the husband’s education level of diploma or less compared to more than diploma; unemployed wives compared to employed; and smoking husbands compared to non-smoking ones.

All articles related to domestic violence during pregnancy in Iran have been included in this study. Therefore, 97 articles were relevant to the research topic from which 35 and 43 papers were excluded due to their repeated topic and non-relevance. After reviewing the abstracts, 4 articles that lacked the required information, desirable qualities, and the inclusion criteria were excluded from the research, and finally 28 articles were included in the meta-analysis (Flow Chart 1).

***Statistical Analysis***

ORs and CIs were calculated for all the articles using the provided data in the agreed tables. To estimate the ORs, the formula OR=$\frac{\mathrm{a*d}}{b*c}$ was used. Combining ORs according to CIs of 95% is based on the weighted average, so that the greater the CIs, the higher the ORs. To combine the results in cases where the studies were homogeneous, the fixed effects model and where they were heterogeneous, random effects model in meta-analysis were used. Index *I*^2^and Cochran test indicated the following results: index *I*^2^ less than 25% indicating low heterogeneity, between 25% to 75% medium non-homogeneous, and more than 75% high heterogeneity. Moreover, to check the amount of heterogeneity between the results, Egger’s test and Funel plot were performed, and to search for publication bias and data analysis, STATA (Version 11.2) software were used. The significance level was set at 0.05.

^1 ^  Odds ratio

^2 ^  Confidence interval

## Results

The studies entered the present meta-analysis were conducted during 2001-2018 and included 25 cross sectional studies, 2 prospective cohort studies and 1 Case-Control studies with a sample of 19243 people and an average of 687 people per study. The findings of this systematic review and meta-analysis are provided for both demographic factors associated with domestic violence against women and stimulating demographic factors for use of violence by men. The specifications of evaluated articles are presented in Table 1.

Demographic factors associated with domestic violence against pregnant women have been studied at 7 levels. Ten studies were examined to determine the relationship between women age during pregnancy and violence during pregnancy. Based on the results of the present meta-analysis, women under the age of 25 years during pregnancy, compared to women who were older than 25 years, were more exposed to violence during pregnancy (OR:1.11); however, this correlation was not significant (Table 2). Four studies were conducted to determine the relationship between marriageable age less than 18 years and domestic violence. The meta-analysis results of these 4 studies did not show a significant relationship between marriageable age under 18 years with domestic violence against pregnant women (OR: 1.14, 95% CI: 0.96, 1.35) (Table 2). 

**Figure 1:**
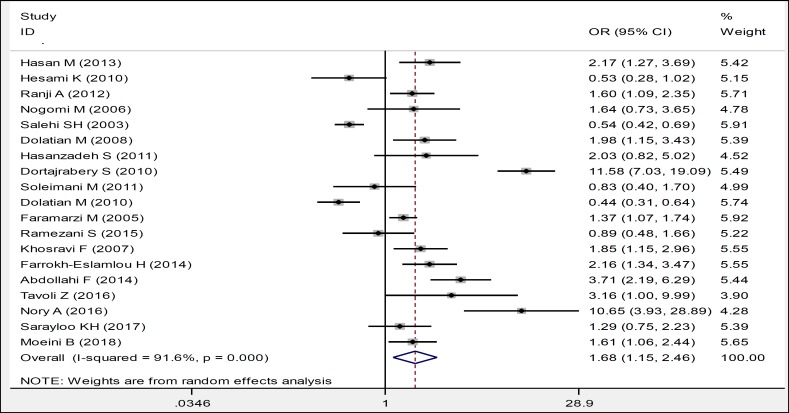
Forest Plots of the Odds Ratio of Maternal Education in Relation to Domestic Violence during Pregnancy and 95% Confidence Interval based on a Random Effect Model in Meta-Analysis. The Midpoint of Each Segment, the Segment Estimating the Odds Ratio, and 95% Confidence Interval in each Study are Demonstrated. Diamond Mark Overall Odds Ratio Is Presented based on the Results of the Meta-Analysis.

Nineteen studies have examined the relationship between maternal education and violence during pregnancy. Ten studies have reported pregnant women with low education as a contributing factor and 2 articles have reported it as a barrier to deal with domestic violence during pregnancy. The ORs of meta-analysis of these studies was OR:1.68, which indicates that low educational level exhibits increased risk of violence against women during pregnancy, and a statistically significant relationship was observed between the educational level of women and domestic violence during pregnancy (OR:1.68, CI 95%: 1.15, 2.46) (Table 2, Figure 1).

Based on the results of the meta-analysis of 18 studies which have investigated the relationship between violence during pregnancy and career of pregnant women, being a housewife during pregnancy would put women at risk of domestic violence (OR:1.04, CI 95%: 0.74, 1.48), but this correlation was not significant (Table 2). To determine the relationship between unintended pregnancy and domestic violence, 12 related studies were found and reviewed. The results of the meta-analysis on the mentioned studies have shown that unintended pregnancy increases the violence against women by their husbands by 1.78 times, which was not statistically significant (Table 2). 

**Figure 2:**
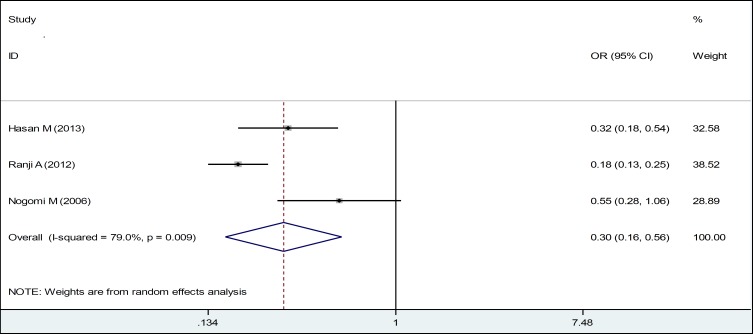
Forest Plots of the Odds Ratio of Women with 3 Children and Less in Relation to Domestic Violence during Pregnancy and 95% Confidence Interval based on a Random Effect Model in Meta-Analysis. The Midpoint of each Segment, the Segment Estimating the Odds Ratio, and 95% Confidence Interval in each Study Are Demonstrated. Diamond Mark Overall Odds Ratio Is Presented based on the Results of the Meta-Analysis.

**Figure 3:**
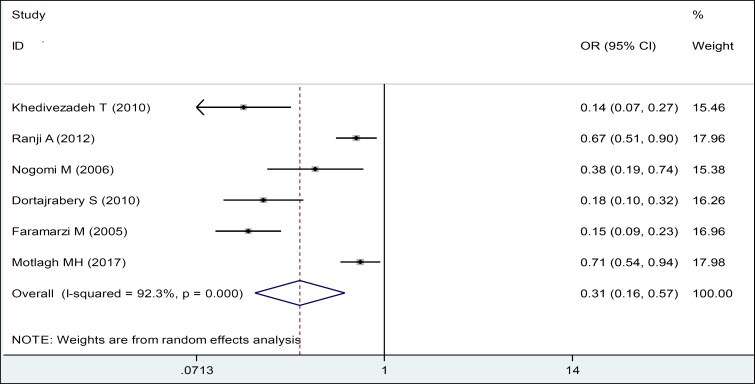
Forest Plots of the Odds Ratio of Inadequate Care during Pregnancy in Relation to Domestic Violence during Pregnancy and 95% Confidence Interval based on a Random Effect Model in Meta-Analysis. The Midpoint of each Segment, the Segment Estimating the Odds Ratio, and 95% Confidence Interval in each Study Are Demonstrated. Diamond Mark Overall Odds Ratio Is Presented based on the Results of the Meta-Analysis

Also, women with 3 children or fewer were less likely to experience domestic violence during pregnancy. In fact, there was a significant inverse relationship between them (Table 2, Figure 2). Meta-analysis of 6 studies in the field adequacy of prenatal care showed that boosting antenatal care attendance has a protective role on violence against women (OR: 0.31, CI 95%: 0.16, 0.57) (Table 2, Figure 3).

**Figure 4:**
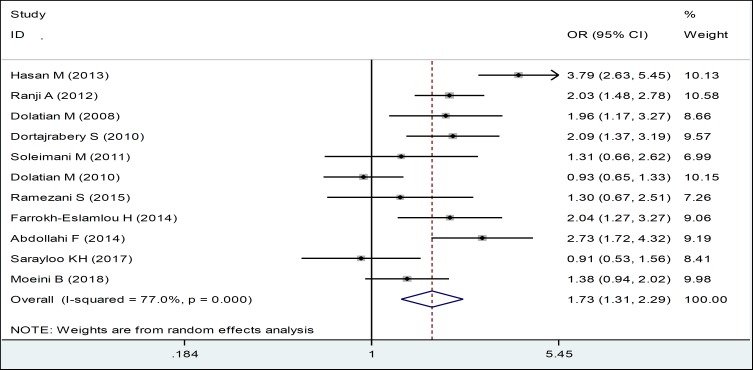
Forest Plots of the Odds Ratio of Spouse's Education Level in Relation to Domestic Violence during Pregnancy and 95% Confidence Interval based on a Random Effect Model in Meta-Analysis. The Midpoint of each Segment, the Segment Estimating the Odds Ratio, and 95% Confidence Interval in each Study are Shown. Diamond Mark Overall Odds Ratio Is Presented based on the Results of the Meta-Analysis.

To study the demographic factors in men which led them to impose violence against their wives during pregnancy, the relationship between violence and level of education, occupation, and smoking in husbands of pregnant women were examined. The results of meta-analysis of 11 studies revealed the ORs of 1.73, indicating that violence against pregnant women increased in husbands with lower levels of education, and this relationship was statistically significant (Table 3, Figure 4).

**Figure 5:**
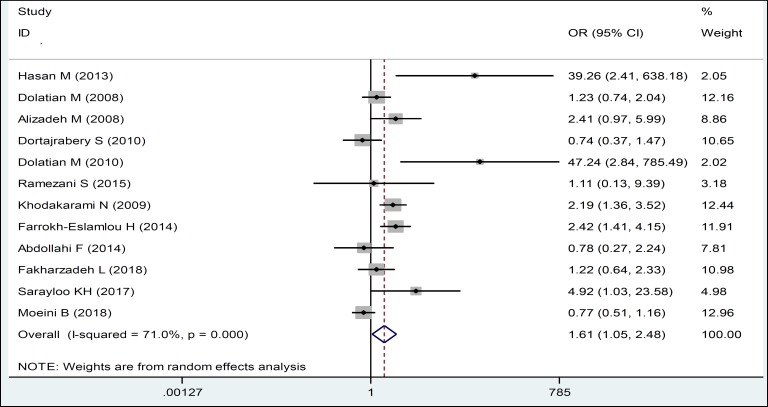
Forest Plots of the Odds Ratio of Unemployed Husbands in Relation to Domestic Violence during Pregnancy and 95% Confidence Interval based on a Random Effect Model in Meta-Analysis. The Midpoint of each Segment, the Segment Estimating the Odds Ratio, and 95% Confidence Interval in each Study Are Demonstrated. Diamond Mark Overall Odds Ratio Is Presented based on the Results of the Meta-Analysis.

**Figure 6:**
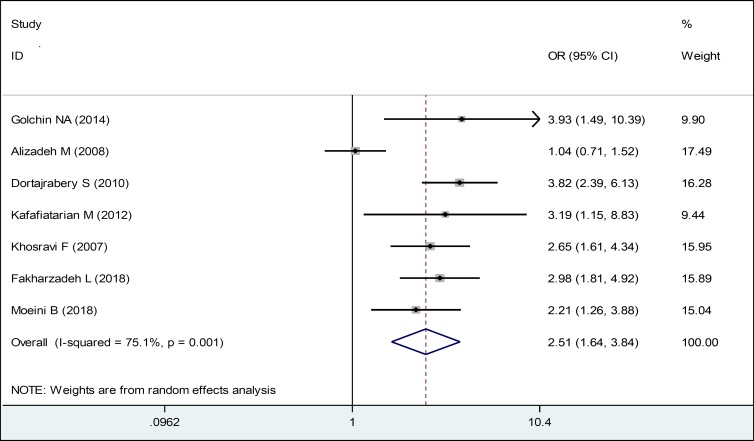
Forest Plots of the Odds Ratio of Smoking Husbands in Relation to Domestic Violence during Pregnancy and 95% Confidence Interval based on a Rrandom Effect Model in Meta-Analysis. The Midpoint of each Eegment, the Segment Estimating the Odds Ratio, and 95% Confidence Interval in Each Study Are Shown. Diamond Mark Overall Odds Ratio Is Presented based on the Results of the Meta-Analysis.

**Figure 7:**
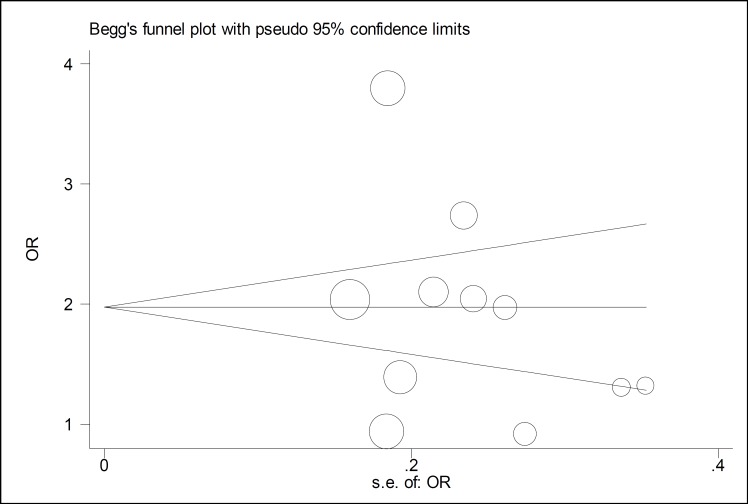
Funnel Plot of the Spouse's Education Level among the Evaluated Studies

The results of this study showed that unemployed husbands showed more domestic violence against their wives during pregnancy. Violence in pregnant women whose husbands were unemployed was 1.61 times more than those with employed husbands. In this regard, there is a statistically significant relationship between them (Table 3, Figure 5). Also, in the present study, a smoking husband caused more domestic violence during his wife’s pregnancy (Table 3, Figure 6). Considering the symmetrical Funnel Plot, it can be concluded that publication bias did not occur in selecting studies and collecting data, whose relationship, even using Egger test (P: 0.465), was not statistically significant (Figure 7).

**Flow Chart 1:**
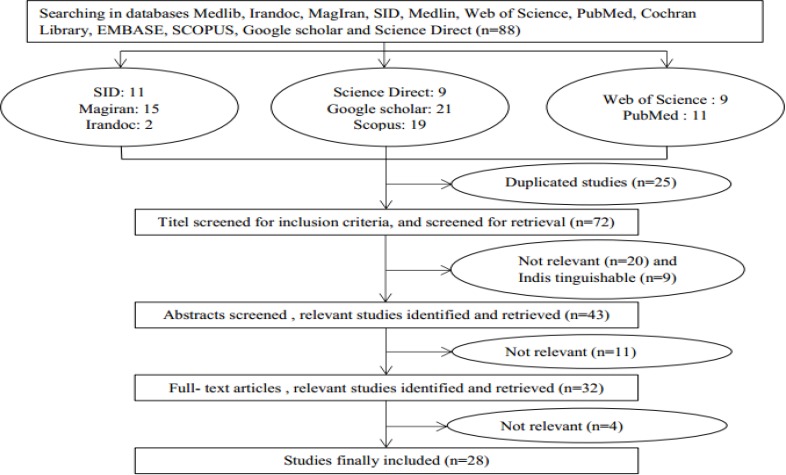
Results of PRISMA Flow of the Systematic Literature Search

**Table 1:** General Characteristics of the Reviewed Articles That Met the Inclusion Criteria

**Author**	**Year, ** **Publication**	**Year,** **Performance**	**Study location**	**Sample size**	**Study design**
Faramarzi et al. (6)	2005	2003-2004	Babol	3275	Descriptive
Ranji & Sadrkhanlo (11)	2012	2007-2008	Urmia	824	Descriptive
Hassan et al. (12)	2014	2009-2010	Mahabad and Miandoab	1300	Descriptive
Dolatian et al. (13)	2008	2007-2008	Marivan	251	Cohort
Salehi & Mehralian (18)	2006	2003	Shahrekord	1600	Descriptive
Hasanzadeh et al. (19)	2011	2009	Ahvaz	300	Descriptive
Soleimani et al. (20)	2012	2009	Tehran	600	Descriptive
Khosravi et al. (21)	2008	2006	Sanandaj	840	Descriptive
Golchin et al. (22)	2014	2013-2014	Golestan	301	Descriptive
Hsami et al. (23)	2010	2007-2008	Marivan	243	Descriptive
Khadivzadeh&Erfania (24)	2011	2005-2004	Mashhad	190	Descriptive
Nojomi et al. (25)	2003	2002	Tehran	406	Descriptive
Dortag-e-Raberi et al. (26)	2010	2007	Tehran	370	Descriptive
Khodakarami et al. (27)	2009	2001-2002	Khoramabad	313	Descriptive
Kafaei-Atrian et al. (28)	2012	2008	kashan	143	Descriptive
Mesdaghinia et al. (29)	2012	2009-2010	kashan	32	Descriptive
Farrokh-Eslamlouet al. (30)	2014	2012	Urmia	313	Descriptive
Abdollahi et al. (31)	2015	2010	Mazandaran	1461	Cohort
Ramezaniet al. (32)	2015	2015	Shahroud	430	Descriptive
Dolatianet al. (33)	2010	2007-2008	Gachsaran	500	Descriptive
Alizadeh et al. (34)	2008	2005-2006	Tabriz	426	Descriptive
Taghizadeh et al. (35)	2015	2014	Tehran	419	Case-Control
Tavoli et al. (36)	2016	2012-2013	Lorestan	230	Descriptive
Motlagh et al. (37)	2017	2015	Six provinces*	2702	Descriptive
Noori et al. (38)	2016	2014	Kolaleh	368	Descriptive
Moeini et al. (39)	2018	2015	Hamadan	1039	Descriptive
Fakharzadeh et al. (40)	2018	2015	Abadan	623	Descriptive
Sarayloo et al. (41)	2017	2012	Minoodasht	300	Descriptive

* Chaharmahal and Bakhtiari, Hamadan, Azarbayjan Gharbi, Khorasan Razavi, Golestan, Hormozgan.

**Table 2:** The Relationship between Demographic Factors of Women with Domestic Violence during Pregnancy via a Meta-Analysis

**Variable**	**Number of study**	**OR (CI 95%)**	**Heterogeneity**	
			**I** ^2 ^ **(%)**	**P value**
Age	10	1.11 (0.68, 1.82)	93.4	0.000
Age at marriage	4	1.14 (0.96, 1.35)	0.0	0.438
Education	19	1.68 (1.15, 2.46)	91.6	0.000
Number of children	3	0.30 (0.16, 0.56)	79.0	0.009
Job	18	1.04 (0.74, 1.48)	83.5	0.000
Unintended pregnancy	12	1.78 (0.82, 3.82)	96.6	0.000
Pregnancy care	6	0.31 (0.16, 0.57)	92.3	0.000

**Table 3:** The Relationship between Demographic Factors of Husbands with Domestic Violence during Pregnancy via a Meta-Analysis

**Variable**	**Number of study**	**OR (95% CI)**	**Heterogeneity**	
			** I** ^2 ^ **(%)**	**P value**
Education	11	1.73 (1.31, 2.29)	77.0	0.000
Job	12	1.61 (1.05, 2.48)	71.0	0.000
Smoking	7	2.51 (1.64, 3.84)	75.1	0.001

## Discussion

This meta-analysis was conducted on demographic factors associated with domestic violence during pregnancy in Iran. The findings of the meta-analysis revealed a significant relationship between violence during pregnancy and spouse's level of education, meaning that ORs of violence in pregnant women who had spouses with lower education level was 1.73 times more than women who had highly educated spouses. It seems that men with higher education level possess better life skills and greater self-awareness to control anger. Babapour et al. (2008), believe that high education level of husbands is a protective factor against violence. Higher education level of husbands brings with itself a better understanding of social and family duties and fair treatment of women and reduces violence (42). In the review of Moafi et al. (2014) in Iran, an inverse correlation was found between education level of husbands and domestic violence during pregnancy (43). In the study of Hassan et al. (2014), the ORs of violence in men with low education level was 1.2 times more than men with higher education level; and education level of men in domestic violence groups was significantly lower than the other group (12). In the study of Hasheminasab et al. (2007), 9.5% of men with low education level and 

1.8% of men with high education level had physical violence towards their partner (44). In a study by Thompson et al. in 2006, a relationship was found between violence and lower educational levels (45). Also, they indicated that the use of violence by men with low education level was more than men with have high education level (8, 18-20, 46). However, in the study conducted by Khedive Zadeh et al. (2011), no significant relationship was found between use of violence and spouse's education level (24). Also, in another study conducted by Horia et al. (2005), there was no significant relationship between violence and spouse's education level (47). In general, increasing the level of education of the husband reduces violence against women.

The findings showed that the chance of facing violence during pregnancy in women with lower education level was more than women with high levels of education. Moreover, a significant correlation was found between violence during pregnancy and education. In the study of Nojomi et al. (2003), women with low education levels experienced violence 1.63 times more than women who were highly educated and this association was statistically significant (25). In another investigation by Faramarzi et al. (2005), women with low education level experienced violence 2.9 times more than women with high education level and this association was statistically significant (6). Also, in the study performed by Jewkes et al. (2002), high educational attainment of women was associated with low levels of violence (48). However, in the study of Khadivzadeh and Erfania (2011), there was no significant relationship between education level of women and violence during pregnancy (24). Also, in study conducted by Soleimani et al. (2012), no significant relationship was found between education level of women and violence during pregnancy (20).

The results showed that the odds of domestic violence against housewife pregnant women were greater than employed women, but the relationship was not significant. Macy et al. (2007), in this study showed there was no significant difference between occupation of women and domestic violence against them (49). But, in the study conducted by Rathora et al. (2002), housewives were more likely to be victims of violence than employed women (50). Also, in the study by Hassan et al. (2014), the possibility of violence among housewives was 1.5 times more than employed women and the relationship was significant (12). Although improving the economic conditions of individuals can influence violence prevention, housewife women have an effective role in creating more favorable mental conditions for their husbands at home, which can lead to a reduction in domestic violence.

Based on the results of this study, the odds of exposure to domestic violence in pregnant women whose husbands were unemployed was 1.61 times more than women with employed husbands. Moreover, a significant relationship was observed between husband's occupation and domestic violence against pregnant women. It seems that unemployed men would commit violence due to financial stress, economic problems, and inability to meet the financial demands of their wives. Bodagh Abadi (2007), had stated that men's unemployment can cause stress and financial problems and because of their presence at home, they may get violent against their wives in case of martial conflicts (1). In the study of Hassan et al. (2014), the odds of unemployed men to commit violence was 1.39 times more likely than employed men (12). In the study of Khosravi et al. (2008), 81.3% of women whose husbands were unemployed experiences violation, while 59.8% of women whose husbands were employed were subjected to violence, and there was a significant correlation between the prevalence of violence and male employment (21). However, in the study of Dolatiyan et al. (2008), there was no significant relationship between husband's occupation and domestic violence against pregnant women (13). 

Marriage age younger than 18 years was one of the contributing factors for exposure of pregnant women to domestic violence. However, in this study, a significant relationship was not found between violence during pregnancy and age of marriage. Perhaps one of the reasons for this finding is low life skills in women and men. In the study conducted by Bagher Zadeh et al. (2008), there was no significant relationship between women's age of marriage and incidence of domestic violence among pregnant women (51). Also, in study of Ranji and Sadrkhanloo (2012), no significant relationship was found between women's age of marriage and violence during pregnancy (11). However, in the study of Shamsi et al. (2012), age of marriage was a contributing factor of gender-based violence among men and there was a significant relationship between violence during pregnancy and women's age of marriage (52). Also, in the study of Khosravi et al. (2008), 65.5% of women younger than 18 years at the time of marriage and 57.7% of women older than 18 years at time of marriage were subjected to violence and significant relationship was found between violence during pregnancy and women's age of marriage (21).

About the role of women's age during pregnancy according to the present meta-analysis, risk of violence during pregnancy in women younger than 25 years was higher than women older than 25 years. However, this association was not statistically significant. In the studies conducted by Ranji and Sadrkhanloo (2012) and Dolatiyan et al (2010), there was no significant correlation between women's age during pregnancy and domestic violence against them. In the study of Ranji and Sadr Khanloo, the possibility of violence in women 26-34 years was 1.65 times more than women younger than the age of 25 years. In the research conducted by Dolatiyan, 58% of women subjected to violence were 23 to18 years old (11, 33). In the study performed by Macy et al. (2007), there was no statistically significant relationship between the age of women during pregnancy and violence during pregnancy (49). 

Evaluating the studies indicated that smoking of men was one of the influencing factors in committing domestic violence against women. The odds of domestic violence against pregnant women by men who smoke was greater than non-smoking men, and this association was statistically significant. In the study performed by Hasheminasab et al. (2007), 13.4% of husbands of abused women and 5.5% of husbands of unviolated women were smoker, and there was a significant relationship between domestic physical violence and smoking (44). In the research of Kafaei Atriyan et al. (2012), the odds of violence in men who used cigarettes, alcohol, or drugs was 3.1 times more than men who did not use them (28). In the study of Hassan Zadeh et al. (2011), the prevalence of violence in smoking men was 28.3% and the greatest amount of violence was done by these men (19). Yanni Karam et al. (2006) and Shamsi et al. (2012), believe that smoking by men is a contributing factor in committing domestic violence against women (52, 53). In the study performed by Hedin and Janson (2007), a significant relationship was found between violence during pregnancy and smoking of men (54).

The results indicated that odds of domestic violence against pregnant women who had fewer children was lower than women with more children, and there was a significant relationship between the number of children and domestic violence against pregnant women. In the study of Ranji and Sadrkhanloo (2012), women who had more children, were exposed to violence 1.83 times more than others (11). In another study conducted by Hassan et al. (2014), women with more than 3 children were subjected to violence during pregnancy 1.25 times more than women with fewer children (12).

In 2005, the World Health Organization has stated that violence, the power to choose, and decision-making lead women to unwanted pregnancy (2). Based on the present meta-analysis, risk of domestic violence in women who had unwanted pregnancy was 1.78 times more than women who had planned pregnancy. However, the relationship was not significant. In the study of Lau (2005), violence was higher among women with unwanted pregnancies, and there was a significant relationship between unintended pregnancy and exposure to domestic violence (55). In the study by Ranji and Sadrkhanloo (2012), prevalence of domestic violence in women who had unwanted pregnancy was 1.84 times higher than in women with unwanted pregnancy (11). In another work done by Goodwin et al. (2000), the domestic violence by a spouse or partner during pregnancy was increased 2.5 times in women with unwanted pregnancies (56). Also, in the study of Moafi et al. (2014), which was conducted as a review in Iran, a direct relationship was found between unplanned pregnancy and an increase in domestic violence during pregnancy (43). However, in the study of Macy et al. (2007), no significant association was found between pregnancy and exposure to domestic violence (49). 

According to the findings of this research, the odds of violence in women who had received adequate care during pregnancy was lower than women who had not received adequate prenatal care or referred to health centers with delay. In the study conducted by Cuningham et al. (2010), women who did not receive prenatal care or attended to health centers with delay were subjected to violence more and there was a significant correlation between violence during pregnancy and prenatal care (57). In the study of Ranji and Sadrkhanloo (2012), the possibility of not receiving prenatal care was 2 times in women who were victims of violence, and it was 2.41 times greater in women who attended to health centers for prenatal care with delay than women who did not experience violence (11). In the another study of Khadivzadeh and Erfania (2011), lack of timely and regular prenatal care for abused women was 7.22 times higher than those women who were not abused (24). In the study conducted by Moafi et al. (2014), there was a direct relationship between inadequate prenatal care and an increase in domestic violence during pregnancy (43).

## Limitation

Limitations of the study were as follow: (1) Lack of a standardized questionnaire in the studies; (2) lack of a standard definition to check the relationship between estimated variables; (3) low quality of some studies which led to their removal from the study; (4) lack of integration or control of environmental confounding variables by using statistical methods in some studies; (5) Heterogeneity between the studies; (6) the low number of studies in some fields of study.

## Conclusion

According to the results of the present meta-analysis, unintended pregnancy, level of education, unemployment, prenatal care, smoking, and number of children were associated with violence during pregnancy. Thus, it appears that by implementing intervention programs and providing special training programs to teach life skills in schools (high schools and colleges), offering family therapy, identifying pregnant women at risk, and modifying the risk factors mentioned above, domestic violence can be reduced in women during pregnancy.
